# Histatin-Based Metallopeptides Target Secondary Biofilms Derived from *Candida albicans* Persister Cells in Recurrent Vulvovaginal Candidiasis

**DOI:** 10.3390/microorganisms14102304

**Published:** 2026-10-10

**Authors:** Gabriela Vieira Silva Zolin, Angela Maione, Marianna Imparato, Antonietta Siciliano, Francesca Esposito, Naiara Maia Pereira, Renan L. de Farias, Saulo Santesso Garrido, Emilia Galdiero, Marco Guida

**Affiliations:** 1 Department of Biology, University of Naples Federico II, 80126 Naples, Italyangela.maione@unina.it (A.M.); francesca.esposito@unicampania.it (F.E.);; 2 Department of Biochemistry and Organic Chemistry, Sao Paulo State University (Unesp), Araraquara 14800-903, Brazil; 3 Department of Experimental Medicine, Luigi Vanvitelli University of Campania, Via Santa Maria di Costantinopoli 16, 80138 Naples, Italy; 4 Department of Chemistry, Pontifical Catholic University of Rio de Janeiro (PUC-Rio), Rio de Janeiro 22451-900, Brazil; renan.farias@puc-rio.br

**Keywords:** antimicrobial peptides, persisters, biofilm, clinical isolates, drug-resistant, *Caenorhabditis elegans*

## Abstract

Biofilm-associated persister cells contribute to the recurrence and treatment failure of *Candida albicans* infections. Here, two Histatin 5-derived peptides (P1 and P2) and their silver(I)-coordinated derivatives (M1 and M2) were evaluated against a vaginal *C. albicans* clinical isolate, focusing on persister-derived secondary biofilms. Amphotericin B-surviving cells regenerated mature biofilms reaching approximately 6.5 log CFU/well after 7 days. At 320 µmol L^−1^, P1, P2, M1, and M2 reduced secondary-biofilm biomass by 68%, 63%, 77%, and 70%, respectively, while M1 and M2 decreased residual metabolic activity to approximately 19% and 18%. Metallopeptides strongly downregulated *SAP1*, *SAP2*, *SAP6*, and *ECE1*, whereas P1 and P2 slightly upregulated *ECE1.* Silver(I) coordination enhanced antifungal and antibiofilm activity but increased host toxicity, highlighting the need to optimize the activity–toxicity balance of these Histatin-derived scaffolds.

## 1. Introduction

*Candida* species are among the major causes of fungal infections, with *Candida albicans* being the predominant species, although infections caused by non-*albicans Candida* species are increasingly reported. As a commensal microorganism of the human microbiota, *C. albicans* can undergo a transition to a pathogenic state under favorable host conditions [1,2]. Current antifungal therapies mainly rely on azoles and polyenes; however, their use is limited by toxicity and the emergence of antifungal resistance [3,4,5]. The increasing prevalence of antifungal resistance, together with the limited availability of selective targets in eukaryotic fungi, highlights the urgent need for new and safer antifungal strategies [6,7,8,9]. As the limitations of conventional agents become more pronounced, exploring innovative classes of molecules has become critical, positioning host-defense peptides as promising candidates. Among these, antimicrobial peptides (AMPs) are small, often cationic peptides that play an important role in host defense by directly inhibiting or killing microorganisms and, in some cases, modulating immune responses. They include lactoferrins, defensins, cathelicidins, and histatins [10,11,12,13]. Histatin 5 (Hst5), a 24-amino-acid peptide naturally present in human saliva, exhibits potent antifungal activity against *C. albicans*. Its predominantly intracellular mechanism involves uptake by fungal cells and subsequent cellular damage associated with oxidative stress and disruption of ion and ATP homeostasis [14,15,16]. However, Hst5 activity can be compromised by environmental factors and proteolytic degradation, while *C. albicans* can employ mechanisms such as protease secretion and AMP efflux to evade its activity [12,14,17,18].

Strategies to improve the antifungal activity and stability of Hst5 include its incorporation into delivery systems and, particularly, structural modification or use of proteolytic fragments that retain antimicrobial activity while reducing susceptibility to degradation [19,20,21,22]. Among these approaches, substitution of lysine residues with arginine has been shown to increase resistance to proteolytic degradation by *C. albicans* proteases and enhance antifungal activity. Moreover, Hst5 derivatives containing arginine substitutions have demonstrated greater inhibition of fungal biofilm formation and viability than the native peptide [23,24].

Thus, the synthesis of a fragment of Hst5 was proposed, composed of the functional domain of the original peptide, whose antifungal activity has already been proven to be retained, named 14 Hst5. In this fragment, a double modification of the lysine residues by arginine was applied, named 14 Hst5 K11,17R, to optimize the anti-*Candida* action and minimize proteolytic degradation. A tryptophan residue was also added to the N-terminal of both peptides to allow fluorescence assays. The primary structure of both peptides is presented in Table 1.

**Figure 1:**
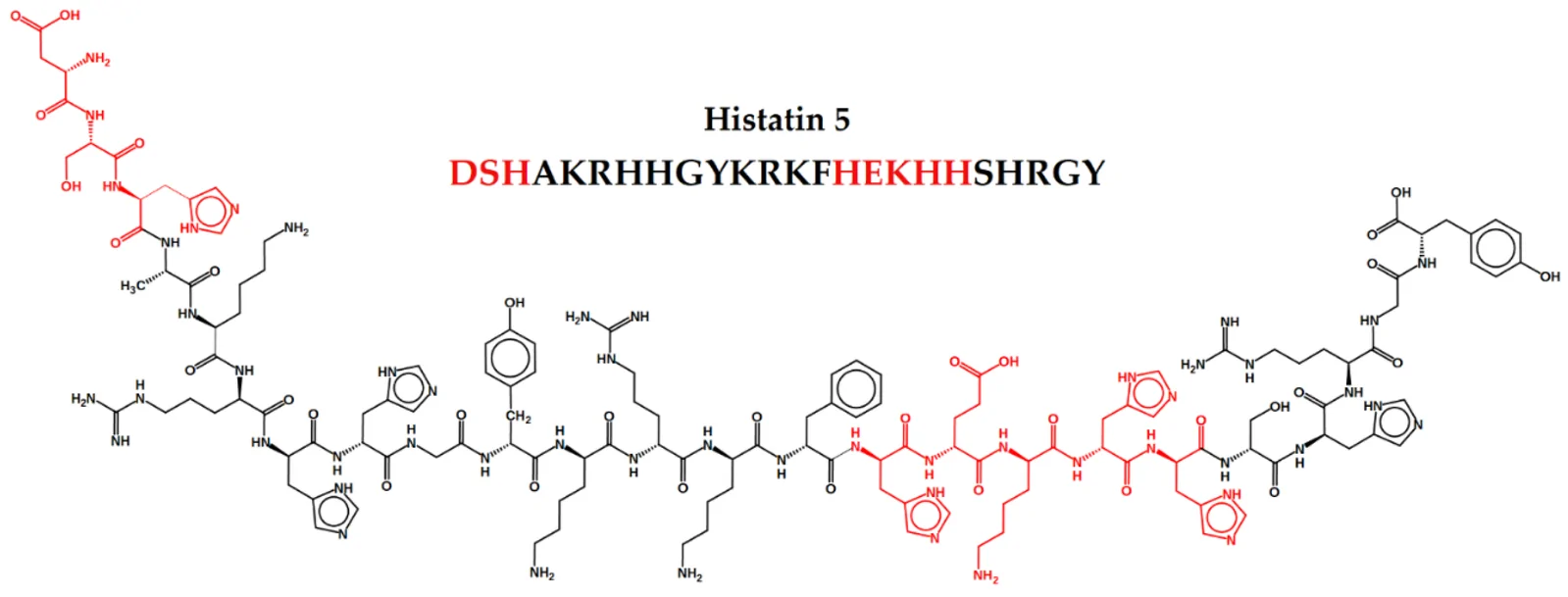
Primary structure of Hst5 with the ATCUN and HEXXH linking motifs, composed of amino acids such as serine, histidine, tyrosine, aspartic acid, and glutamic acid [21].

Another alternative to improve antimicrobial action involves the coordination of peptides with metal ions. Moderately bioactive molecules may show an increased activity profile when associated with metal ions. This characteristic is linked both to a synergistic effect between the ligand and the metal in the metallodrug, as well as to the intrinsic properties of the metal centers [25,26,27]. The ability of Histatin 5 to coordinate with metal ions is favored by the presence of amino acids such as serine, histidine, glutamic acid, aspartic acid, and tyrosine, which are present in large quantities in the peptide chain, in addition to known binding motifs such as ATCUN (amino-terminal copper and nickel binding unit) and HEXXH, as shown in Figure 1 [28,29,30]. Several studies have already been conducted to verify the activity and stability of the peptide when coordinated to transition metal ions Cu(II), Zn(II), Fe(III), Ni(II), and Co(II), and alkali and alkaline earth metals such as Ca(II) and Mg(I), obtaining varied results [14,31,32,33,34,35].

Silver ions [Ag(I)] possess broad antimicrobial activity, acting through multiple mechanisms, including disruption of membrane integrity, inhibition of essential enzymes and electron transport, and interference with DNA replication [36,37,38]. Based on these properties, Ag(I) was coordinated with 14 Hst5 and 14 Hst5 K11,17R to generate peptide–silver complexes with enhanced potential against *C. albicans*. Previous circular dichroism, fluorescence spectroscopy, and high-resolution mass spectrometry analyses confirmed the formation of stable 1:1 peptide/Ag(I) coordination complexes.

Beyond the intrinsic activity of these compounds, a major obstacle in recurrent candidiasis is the survival of tolerant subpopulations within biofilms. Persister cells (PCs) represent a major contributor to the recalcitrance of chronic biofilm-associated infections and pose a significant public health and safety threat. Persistence is a transient phenotypic state in which a small subpopulation of genetically identical cells survives antimicrobial treatment by entering dormancy, despite remaining genetically susceptible to antibiotics. Following treatment, persisters can repopulate and regenerate a new microbial population containing a similar fraction of tolerant cells. The ability of biofilms to harbor and protect persister cells is considered one of the main factors underlying the difficulty of eradicating chronic biofilm infections [39]. Biofilm-associated persister cells are increasingly recognized as key contributors to the persistence and recurrence of vulvovaginal candidiasis (VVC), owing to their ability to survive antifungal treatment and regenerate biofilms. Previously, we demonstrated [40] that the antimicrobial peptide gH625 synergizes with conventional antifungals to eradicate *C. albicans* biofilms originating from amphotericin B-tolerant persister cells. We also showed that oteseconazole (VT-1161), a novel tetrazole antifungal, effectively eliminates secondary biofilms generated by persister cells in a fluconazole-resistant vaginal isolate of *C. albicans*. Collectively, these findings support the development of therapeutic strategies specifically targeting biofilm-associated persister populations to reduce treatment failure and disease recurrence [41].

The present study investigated the antibiofilm activity of the antimicrobial peptides 14 Hst5 and 14 Hst5 K11,17R, alone or conjugated with Ag(I), against a sessile and PC-derived biofilm of a *C. albicans* clinical isolate. Their ability to inhibit hyphal switching and modulate the expression of the ECE1 gene, which encodes the virulence factor candidalysin, as well as the SAP1, SAP2, and SAP6 genes, which encode aspartyl proteinases involved in tissue degradation, adhesion, and nutrient acquisition, was evaluated. In addition, the *Caenorhabditis elegans* model was employed to assess their in vivo antifungal efficacy and their potential to reduce the risk of resistance development.

## 2. Materials and Methods

### 2.1. Solid-Phase Peptide Synthesis and Purification and Metallopeptide Formation

The peptide fragments 14 Hst5 (WKRKFHEKHHSHRGY) (P1) and 14 Hst5 K11,17R (WRRKFHERHHSHRGY) (P2) were synthesized using the Merrifield solid-phase strategy (1963), starting from a Wang-type resin, according to the Fmoc/*t*-Butyl protocol. The amino acids were then solubilized in dimethylformamide (DMF) and dichloromethane (DCM) 1:1, and the coupling agents *N*,*N*-diisopropylcarbodiimide (DIC) and *N*-Hydroxybenzotriazole (HOBt) were used with a three-fold molar excess. Each coupling was verified using the ninhydrin test, according to the Kaiser protocol, to detect free terminal amino groups [42], and the deprotection of each α-amino group was carried out in a 20% (*v*/*v*) 4-methylpiperidine solution in DMF. The cleavage procedure was conducted using a solution composed of 95% (*v*/*v*) trifluoroacetic acid (TFA), 2.5% deionized water, and 2.5% triisopropylsilane (TIS), under slight agitation for 3 h at room temperature. Subsequently, the peptides were extracted with 10% (*v*/*v*) acetic acid.

The peptides were purified through a HPLC system in semi-preparative mode, using a Zorbax Eclipse XDB C18 reversed-phase column (9.4 × 250 mm and 5 µm). The chromatographic conditions consisted of solvents A (0.045% TFA in H_2_O) and B (0.036% TFA in ACN), a gradient of 0.33% min^−1^ of solvent B for 90 min and a flow rate of 2 mL min^−1^, and detection wavelengths at 220 and 280 nm. Qualitative analysis was performed in analytical mode using a Shimadzu LC-10A/C-47A separation system coupled to a Shimadzu LC-10A/C-47A UV-Vis detector (Shimadzu, Kyoto, Japan), with a Waters Symmetry C18 column (2.1 × 150 mm and 5 µm). For the analytical mode, solvents A (0.045% TFA in H_2_O) and B (0.036% TFA in ACN) were utilized, with gradients ranging from 5% to 95% of solvent B for a period of 30 min under a flow rate of 0.6 mL min^−1^. The peptides obtained were characterized and identified by a HPLC system coupled to a mass spectrometer with electrospray ionization in positive mode, on a Bruker Amazon mass spectrometer with an ion-trap analyser (Bruker, Billerica, MA, USA) [43].

The coordination of peptides with Ag(I) ions was achieved freely, by adding a silver nitrate (AgNO_3_, 99.99%) solution to a solution of each peptide, both at the same concentration, resulting in metallopeptide ratios of 1:1, accordingly to previous studies (M1 and M2). The reaction proceeded for 30 min at room temperature, in a dark environment. The metallopeptide formation, at a concentration of 10 µmol L^−1^, was confirmed by circular dichroism spectra, obtained utilizing Jasco J-815 equipment (Jasco, Hachioji, Japan), comprising accumulation of 12 scans. The wavelengths ranged from 200 to 250 nm, with recordings made at intervals of 0.1 nm, and scanned at a speed of 100 nm min^−1^. The metallopeptide mass confirmation was analyzed using a Bruker Daltonics MALDI Ultraflextreme spectrometer equipped with a TOF/TOF analyzer (Bruker Corporation, Bruker Daltonics, Bremen, Germany), with MS and MS/MS data acquisition (reflector mode). The coordination compounds were prepared in a 1:1 (*v*/*v*) ratio with a 10 mg/mL matrix of 2,5-dihydroxybenzoic acid (DHB).

### 2.2. Candida Growth Conditions

Two *C. albicans* strains were tested: the reference *C. albicans* ATCC 90028 and a clinical isolate identified as *C. albicans* 123D (C19) associated with vaginal infection from a VVC patient, and kept in our collection in the Hygiene laboratory.

They were grown in a YPD (yeast extract 2%, bactopeptone 1%, and dextrose 2%) or RPMI (Roswell Park Memorial Institute medium) (Thermo Fisher Scientific Inc., Waltham, MA, USA) medium at 37 °C standardized to 1 × 10^3^ CFU mL^−1^ for planktonic assays and 1 × 10^6^ CFU mL^−1^ for biofilm assays.

### 2.3. HaCaT Keratinocyte Cell Culture and Cytotoxicity Assay

Human immortalized keratinocyte cells (HaCaT) were used as an in vitro model of epidermal cells. HaCaT cells were cultured in Dulbecco’s Modified Eagle Medium (DMEM) supplemented with 10% fetal bovine serum (FBS), 1% L-glutamine, and 1% penicillin/streptomycin. Cells were maintained at 37 °C in a humidified atmosphere containing 5% CO_2_. The culture medium was replaced every two to three days, and cells were subcultured when they reached approximately 80–90% confluence.

The cytotoxicity of peptides P1 and P2 and metallopeptides M1 and M2 was evaluated using a cell viability assay. Briefly, cells were seeded in 96-well plates at a density of 1 × 10^4^ cells/well in complete culture medium and allowed to adhere overnight at 37 °C in a humidified atmosphere containing 5% CO_2_. After incubation, cells were treated with increasing concentrations of each compound, ranging from 10 to 320 µmol L^−1^, prepared in complete culture medium.

Untreated cells were used as negative controls and considered as 100% viability [44]. After treatment, cell viability was assessed using a colorimetric assay according to the manufacturer’s instructions. Absorbance was measured using a microplate reader, and the percentage of viable cells was calculated by comparing the absorbance values of treated cells with those of untreated control cells, according to the following formula:

$$\mathrm{Cell viability}\left(\%\right)=\frac{\mathrm{Absorbance of treated cells}}{\mathrm{Absorbance of untreated control cells}}\times 100$$

### 2.4. Antifungal Susceptibility Assay in Planktonic Cells

Minimum inhibitory concentration (MIC) and minimum fungicidal concentration (MFC) assays of peptides and metallopeptides against *Candida* strains were performed according to the Clinical and Laboratory Standards Institute (CLSI) broth microdilution reference method for yeasts [45]. Peptides and metallopeptides were tested at concentrations ranging from 10 to 640 μmol L^−1^ in sterile 96-well microplates containing yeast suspensions adjusted to 1 × 10^3^ cells mL^−1^. Plates were incubated at 37 °C for 24 h. MIC values were defined as the lowest concentration of each compound resulting in total growth inhibition, as determined by measuring absorbance at 590 nm using a microplate reader. MFC values were determined by subculturing aliquots from wells showing no visible growth onto appropriate agar plates (YPD agar plate); the MFC was defined as the lowest concentration resulting in a 99.9% reduction in the initial inoculum, evidenced by the absence of colony growth after incubation.

### 2.5. Germ Tube Formation

The ability of *C. albicans* to undergo yeast-to-hypha transition was evaluated using the germ tube formation assay, as previously described by Pericolini et al. [46]. Briefly, a single colony of C19, recovered from a 24 h YPD agar culture, was inoculated into 0.5 mL of RPMI 1640 medium supplemented with 10% fetal bovine serum (FBS; Thermo Fisher Scientific, Cleveland, OH, USA) in 24-well culture plates. Peptides and metallopeptides, dissolved in RPMI 1640 medium, were added at a final concentration of 160 μmol L^−1^. Control wells received medium without compounds. Plates were incubated at 37 °C for 4 h to induce germ tube formation. Following incubation, germ tube development was assessed using a JuLI™ Stage inverted microscope (NanoEntek, Seoul, Republic of Korea) at 4× magnification.

### 2.6. Antifungal Susceptibility Assay in Biofilms

To evaluate the biofilms’ inhibition, cell suspensions, at 10^6^ cell mL^−1^, in RPMI 1640 were placed in 96-well polystyrene microtiter plates (100 μL per well), with peptides and metallopeptides (range 20–160 μmol L^−1^), and incubated for 24 h at 37 °C as reported previously [47]. After incubation, the medium was removed and the biofilms were washed with phosphate-buffered saline (PBS 0.01 M) to remove non-adherent cells and air-dried before adding 150 μL of 1% *w*/*v* crystal violet (CV) to each well in order to quantify the biofilm-forming cells. The optical density of stained adherent cells was determined at 570 nm with an automatic microplate reader.

To investigate the biofilm eradication action of peptides and metallopeptides, the cells were allowed to adhere to the plates for 24 h and washed with PBS 0.01 M before been incubated with the peptides, at the same concentrations, for another 24 h. The biofilms’ eradication was quantified using a crystal CV assay.

### 2.7. Detection of Biofilm Persister Cells and Eradication Ability of Peptides and Metallopeptides

The detection of persister cells within *C. albicans* clinical isolate biofilms was performed as previously described [40], with minor modifications. Briefly, 24 h mature biofilms (B1), generated as described above, were exposed to amphotericin B (AmB) diluted in RPMI 1640 medium and buffered to pH 7.0 with 0.165 M morpholinopropanesulfonic acid (MOPS; Sigma-Aldrich, St. Louis, MO, USA). AmB was tested at concentrations ranging from 5 to 200 μg mL^−1^, and biofilms were incubated for 24 h at 37 °C. Following treatment, biofilms were thoroughly washed with phosphate-buffered saline (PBS) to remove non-adherent and dead cells. The remaining biofilm was mechanically disrupted by scraping and vigorous vortexing for 30 s. Persister cells were quantified by plating serial dilutions and enumerating surviving colonies, with results expressed as log CFU per well. To generate secondary biofilms (B2), persister cells surviving AmB treatment were allowed to regrow in RPMI 1640 medium for 7 days under static conditions at 37 °C. During this period, 50 μL of fresh medium was added to each well every 24 h, and every 48 h the wells were gently washed with 0.01 M PBS and replenished with fresh medium. Biofilm viability and the absence of planktonic contamination were monitored every 48 h by subculturing aliquots of the supernatant collected from the wells onto agar plates and evaluating colony growth. After 7 days, mature persister-derived biofilms were washed with 0.01 M PBS and exposed to peptides and metallopeptides at concentrations ranging from 80 to 320 μmol L^−1^. Following incubation for 24 h at 37 °C, biofilm eradication was quantified using the CV to detect the residual biomass, and with XTT to detect the residual metabolic activity. Additionally, the viability of C19 secondary biofilm after treatment with a sub-MIC of 160 µmol L^−1^ of peptides (P1 and P2) and metallopeptides (M1 and M2) was evaluated by fluorescent microscopy observation [48]. Briefly, after treatment, the secondary biofilms were gently washed twice with PBS to remove non-adherent cells and residual compounds. The adherent biofilm cells were then stained with 5 µM SYTO 9 fluorescent dye and incubated for 30 min at room temperature in the dark. Excess dye was removed by washing with PBS, and the biofilms were observed using a Zeiss Axioskop fluorescence microscope equipped with an Axiocam MRc5 digital camera and Axiovision 4.7 software (Carl Zeiss, Jena, Germany). The process was explained by a schematic flowchart (Figure S1).

### 2.8. PCR Analysis

To evaluate the modulatory effect of the peptides P1 and P2 and the metallopeptides M1 and M2 on the expression of selected virulence-related genes, secondary biofilms derived from *C. albicans* C19 persister cells were analyzed, as previously described [49]. Briefly, mature primary biofilms of *C. albicans* C19 were treated with amphotericin B to select persister-like surviving cells. These cells were then recovered and allowed to regrow on polystyrene surfaces to generate secondary biofilms. After, samples were treated or not with each peptide or metallopeptide at a final sub-MIC of 160 µmol L^−1^. Untreated secondary biofilms were used as controls. After incubation, biofilm-associated *C. albicans* cells were carefully recovered, washed with PBS, and processed for total RNA extraction using the Direct-zol™ RNA Miniprep Plus Kit (Zymo Research, Irvine, CA, USA), according to the manufacturer’s protocol. Subsequently, 1 µg of purified RNA was reverse-transcribed into cDNA using a Bio-Rad reverse transcription kit (Bio-Rad, Milan, Italy). Quantitative real-time PCR analysis was performed using an AriaMx Real-Time PCR system (Agilent Technologies, Inc., Milan, Italy). For each reaction, 100 ng of cDNA was used.

The amplification protocol consisted of an initial denaturation step at 95 °C for 10 min, followed by 40 amplification cycles of 95 °C for 15 s and 60 °C for 1 min. A final step at 95 °C for 15 s was included, followed by melting curve analysis from 60 to 95 °C to confirm the specificity of the amplified products. The primers used for *SAP1*, *SAP2*, *SAP6*, and *ECE1* were those previously reported [50] and shown in Table S1. Gene expression levels were calculated using the comparative Ct method, 2^−ΔΔCt^, with *ACT1* selected as the reference gene for normalization. Data are expressed as relative fold change compared with the untreated secondary biofilm control.

### 2.9. C. elegans Toxicity Assay

The toxicity assay was conducted on *Caenorhabditis elegans* nematodes, a wild-type N2 Bristol variant strain, according to modified ASTM E2172-01 (2014) [51]. The *C. elegans* strain was maintained on Nematode Growth Medium (NGM) agar plates seeded with *Escherichia coli* (uracil-deficient OP50 strain) as the standard food source. Age-synchronized populations were obtained by treating gravid adults with 10% Clorox solution (10 g/L NaOH, 10.5 g/L NaOCl), through several washes with M9 medium (2.2 mM KH_2_PO_4_, 4.2 mM Na_2_HPO_4_, 85.6 mM NaCl, 1 mM MgSO_4_), to isolate embryos. These were subsequently allowed to hatch and develop on NGM plates seeded with *E. coli* OP50 at 20 °C until they reached the L1 larval stage. For survival assays, nematodes synchronized at the L1 larval stage (10–15 organisms for well) were transferred to 24-well microplates containing 500 μL of peptide solutions, each with a final concentration of 160 μmol L^−1^ or the corresponding untreated control. Plates were incubated at 20 °C, and nematode survival was monitored every 24 h over a 72 h period by scoring live and dead organisms. The test was considered not valid if the survival rate in the control group was below 90%.

### 2.10. C. elegans Infection Assay

*Escherichia coli* OP50 was grown overnight in modified LB broth, whereas C19 was cultured overnight in Tryptic Soy Broth (TSB, 1×) supplemented with 1% (*w*/*v*) glucose at 37 °C under constant shaking (200 rpm). Following incubation, cell densities were determined spectrophotometrically at 590 nm, and microbial suspensions were adjusted to a final concentration of 1 × 10^6^ CFU/mL. The *C. elegans* infection assay was adapted from the ASTM E2172-01 [51] toxicity protocol with minor modifications. Nematodes were recovered from NGM agar plates, incubated for three days at 20 ± 2 °C, using K-medium, and treated with a freshly prepared 10% Clorox solution (10 g/L NaOH, 10.5 g/L NaOCl) to eliminate adult and isolate eggs. The eggs were washed several times by centrifugation with M9 buffer (2.2 mM KH_2_PO_4_, 4.2 mM Na_2_HPO_4_, 85.6 mM NaCl, and 1 mM MgSO_4_) to completely remove residual bleach. Purified eggs were transferred onto NGM agar plates and incubated in the dark at 20 ± 2 °C for 16–18 h to obtain synchronized L1 larvae. After hatching, L1 larvae were collected from NGM plates and washed thoroughly with M9 buffer to remove external microorganisms, ensuring that subsequent survival analyses reflected internal infection only. Approximately 15 synchronized L1 larvae were transferred into each well of sterile multi-well plates containing 500 μL of K-medium. Control wells contained *E. coli* OP50 (1 × 10^6^ CFU/mL) as the food source, whereas infection wells contained C19 (1 × 10^6^ CFU/mL). The infected groups were treated with the tested antimicrobial peptides (P1 and P2) or their silver(I) conjugates (M1 and M2). Plates were incubated in the dark at 20 ± 2 °C, and nematode survival was monitored every 24 h for up to 3 days. Dead organisms were scored daily based on the absence of spontaneous movement and failure to respond to gentle mechanical stimulation. All experimental groups were assayed simultaneously in at least three independent experiments.

## 3. Results

### 3.1. Peptide Synthesis and Metallopeptide Formation

**Figure 2:**
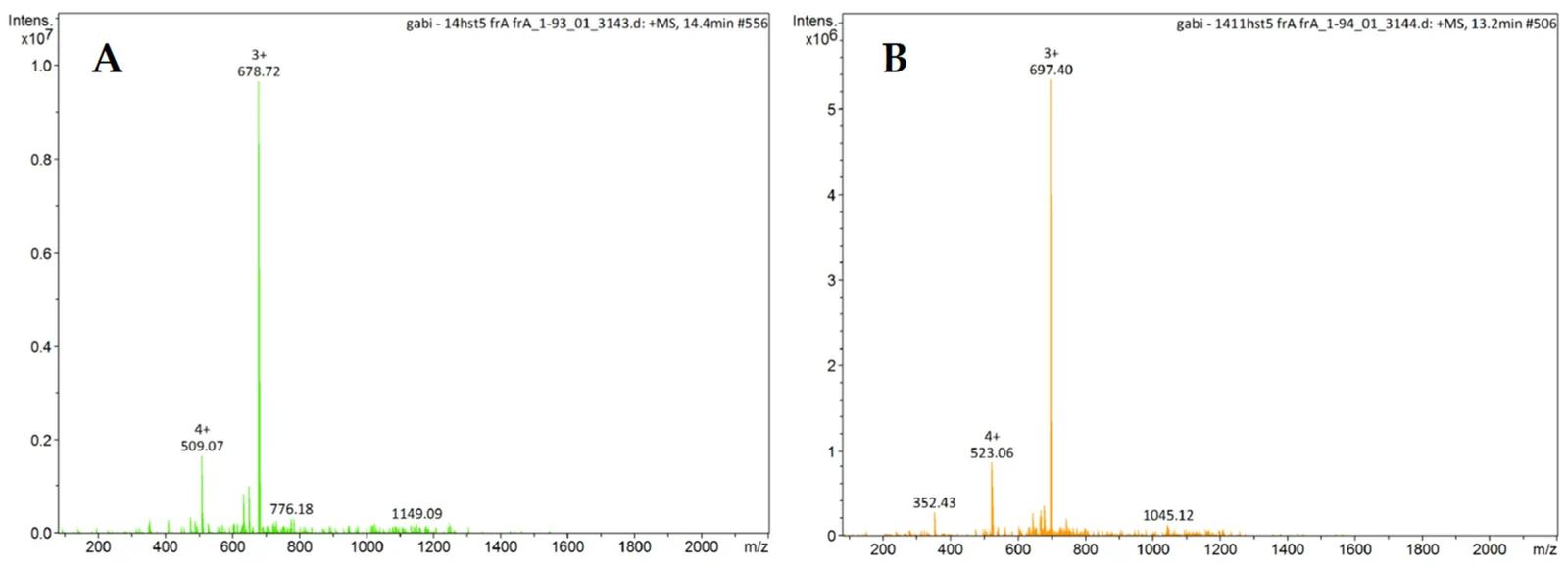
Mass spectrum obtained for peptides (**A**) 14 Hst5 and (**B**) 14 Hst5 K11,17R, after purification, indicating that pure peptides were obtained and confirming their molecular masses.

Two Histatin 5-derived peptides, 14 Hst5 (P1) and the modified analog 14 Hst5 K11,17R (P2), were successfully synthesized and purified for subsequent biological evaluation. Both peptides contained a C-terminal tryptophan residue to facilitate spectroscopic detection. Following purification by reverse-phase HPLC, each peptide exhibited a purity greater than 95%. Electrospray mass spectrometry further confirmed the identity of the synthesized peptides, yielding experimental molecular masses and *m*/*z* values that closely matched the theoretical predictions (Figure 2, Table 2). The excellent agreement between the calculated and experimental molecular masses demonstrates the successful synthesis and high purity of both peptide analogs.

The coordination between the obtained peptides with silver ions, conducted in solution at an equimolar concentration of 1:1, resulted in the metallopeptides 14 Hst5–Ag (M1) and 14 Hst5 K11,17R–Ag (M2).

**Figure 3:**
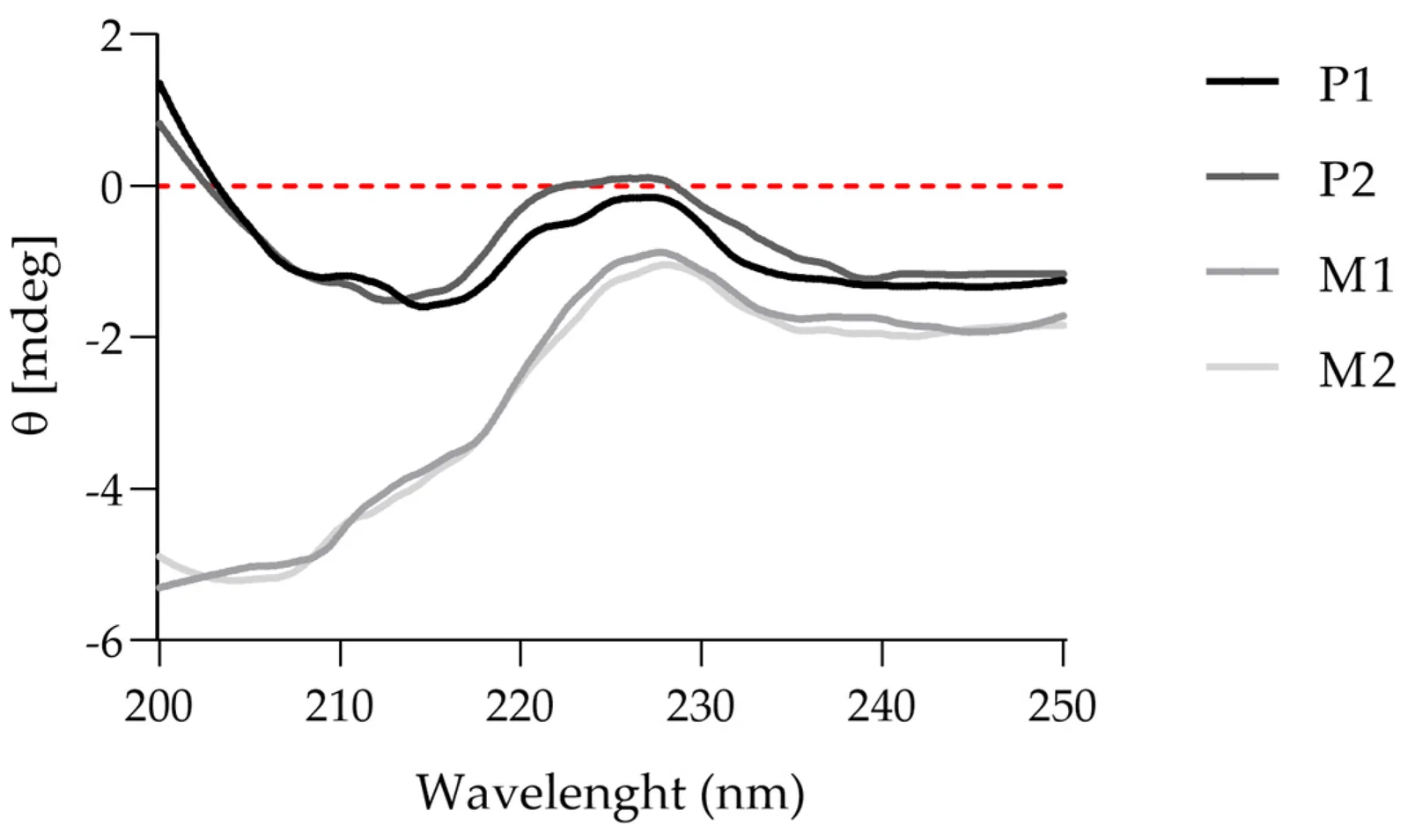
Circular dichroism spectra, from 200 to 250 nm, at intervals of 0.1 nm and a speed of 100 nm min^−1^. Peptides and metallopeptides were at a concentration of 10 µmol L^−1^, smoothed over 50 points.

Based on the CD spectra, as illustrated in Figure 3, P1 and P2 display negative bands at wavelengths approximately between 205–208 and 215–220 nm, and a positive maximum band is observed around 200 nm, characterizing both peptides with an α-helical structure. The alteration in secondary structure is prominently illustrated in the CD profiles of the metallopeptides. The α-helical structure is absent in the metallopeptides’ profiles; in that case, presenting minimum bands at 200 nm and a maximum band between 220 and 230 nm, both metallopeptides assume a random secondary structure, as Ag(I) coordination by amino acid side chains modifies the ligands’ spatial conformation, changing the entire compound’s structure.

**Figure 4:**
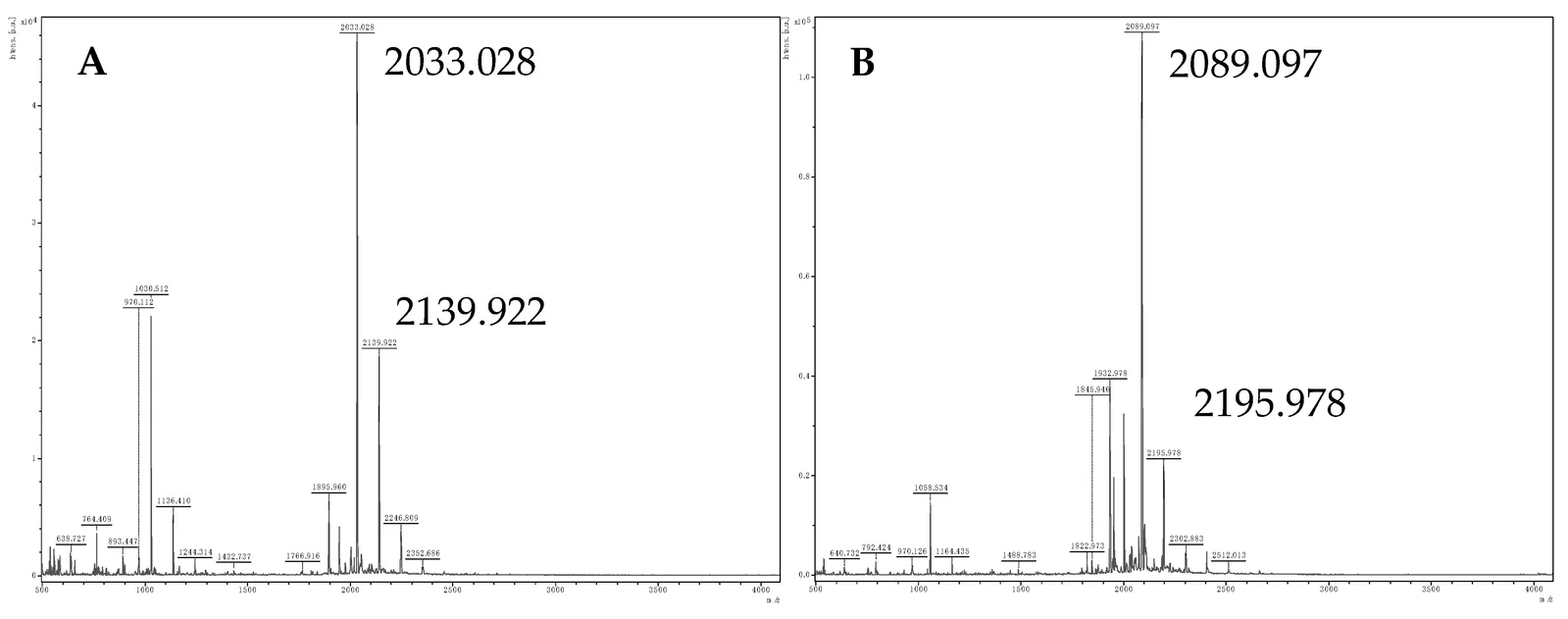
MALDI-TOF mass spectra presenting (**A**) 14 Hst5–Ag (2139.92 g mol^−1^) and (**B**) 14 Hst5 K11,17R–Ag (2195.98 g mol^−1^).

The Matrix-Assisted Laser Desorption/Ionization Time-of-Flight Mass Spectrometry (MALDI-TOF MS) technique, presented in Figure 4, allowed the determination of the metallopeptides’ molar mass without fragmentation, precisely confirming the molar coordination ratio between the peptides and the metal.

As shown in Table 3, the experimentally obtained molecular masses are consistent with the theoretical values calculated for a 1:1 peptide/Ag(I) molar ratio for both metallopeptides; therefore, it can be stated that coordination occurs between one peptide molecule and one Ag(I) molecule.

### 3.2. Cytotoxicity Assessment

**Figure 5:**
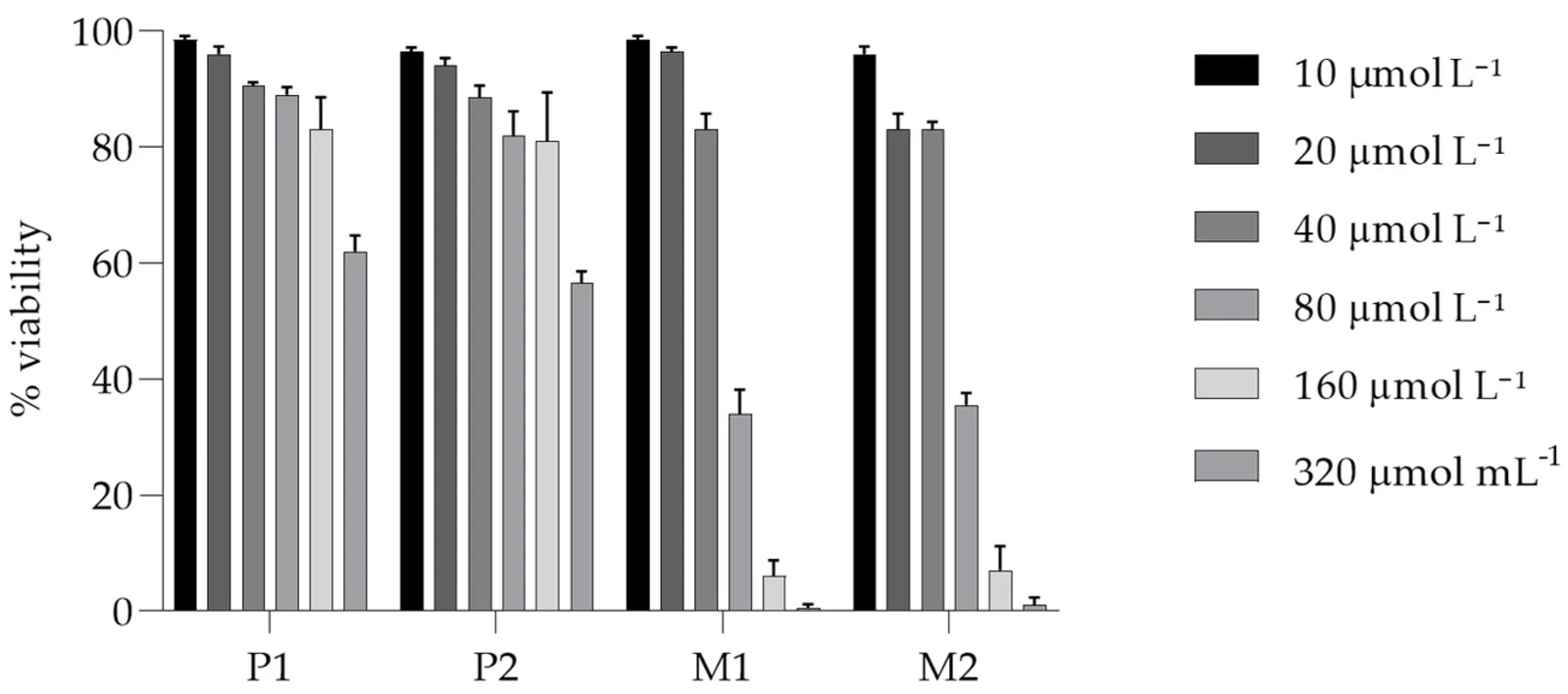
Cytotoxicity of peptides (P1, P2) and metallopeptides (M1, M2) on HaCaT human keratinocytes at a concentration range of 10 to 320 µmol L^−1^. Cell viability is expressed as a percentage relative to untreated control cells and reported as mean ± standard deviation.

The cytotoxicity of peptides P1 and P2 and metallopeptides M1 and M2 was evaluated over a concentration range of 10–320 µmol L^−1^. As shown in Figure 5, P1 and P2 displayed a more favorable cytocompatibility profile than the corresponding metallopeptides. Cell viability remained above 80% up to 160 µmol L^−1^ for both peptides and decreased moderately at 320 µmol L^−1^, reaching approximately 60%. Conversely, M1 and M2 exhibited a marked concentration-dependent cytotoxic effect. Although cell viability remained relatively high at concentrations up to 40 µmol L^−1^, a pronounced decrease was observed from 80 µmol L^−1^ onwards. At 160 µmol L^−1^, viability decreased to approximately 35% for M1 and below 10% for M2, while exposure to 320 µmol L^−1^, corresponding to their antifungal MIC, resulted in almost complete loss of HaCaT cell viability. These findings indicate that Ag(I) coordination enhanced antifungal activity but was associated with substantially increased cytotoxicity at higher concentrations.

### 3.3. Minimal Inhibitory Concentration (MIC) and Minimum Fungicidal Concentration (MFC)

The antifungal activity of the tested peptides and metallopeptides was evaluated against the reference strain *C. albicans* ATCC 90028 and the clinical isolate *C. albicans* C19 (Table 4). The peptides 14 Hst5 and 14 Hst5 K11 showed inhibitory activity against *C. albicans* ATCC 90028, with MIC values of 640 µmol L^−1^. However, no fungicidal activity was detected at the highest concentration tested, as indicated by MFC values > 640 µmol L^−1^. The clinical isolate *C. albicans* C19 appeared less susceptible to the peptides, since both 14 Hst5 and 14 Hst5 K11 showed MIC and MFC values > 640 µmol L^−1^.

Conversely, the metallopeptides 14 Hst5-Ag and 14 Hst5-K11,17R-Ag displayed greater antifungal activity against both strains. For *C. albicans* ATCC 90028, both metallopeptides showed MIC values of 320 µmol L^−1^ and MFC values of 640 µmol L^−1^. The same activity profile was observed against *C. albicans* C19, with MIC and MFC values of 320 and 640 µmol L^−1^, respectively.

### 3.4. Hyphal Growth

**Figure 6:**
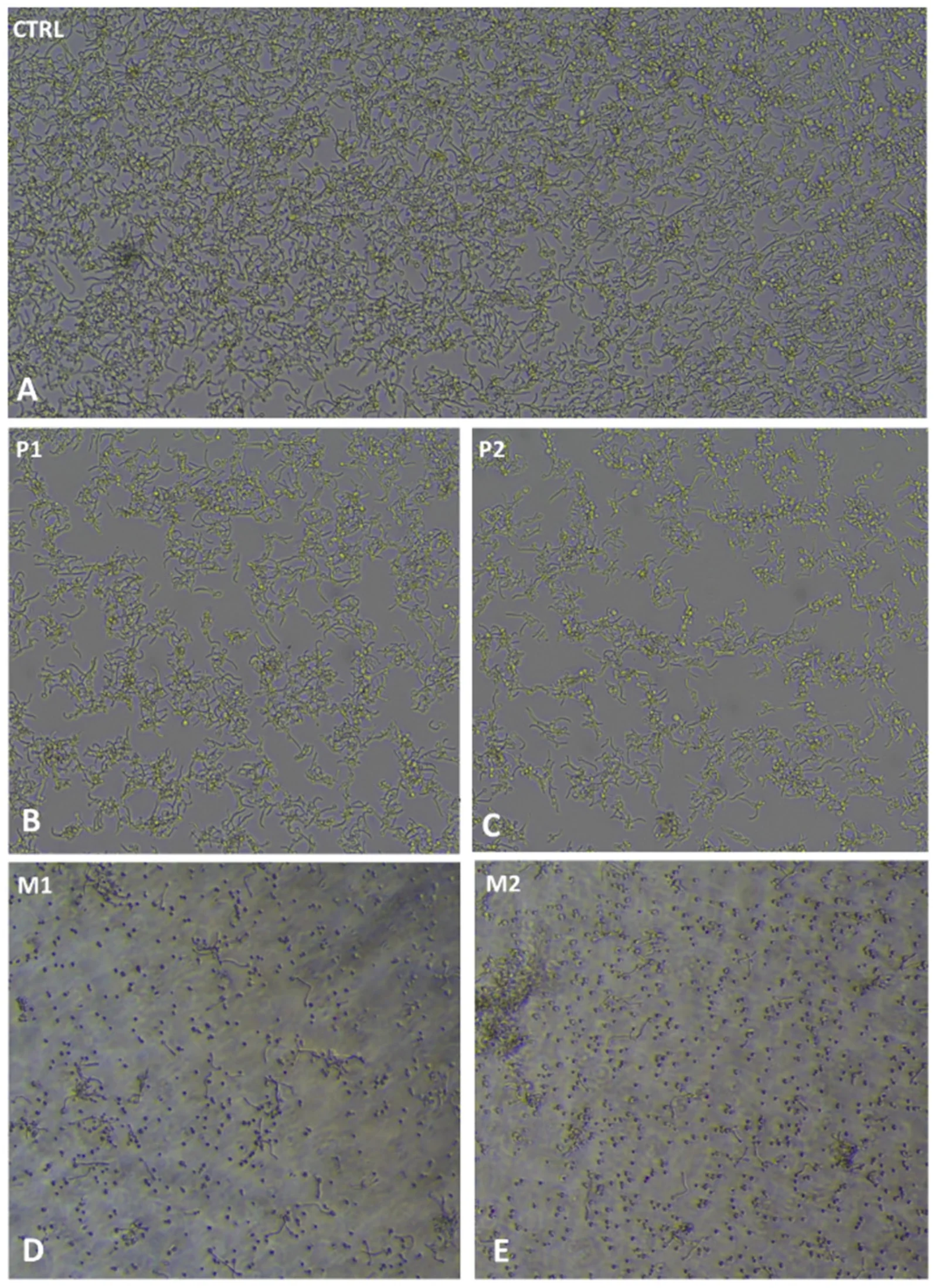
Representative microscopic images of *C. albicans* C19 cells under germ tube-inducing conditions, untreated (**A**) or treated with peptides P1 (**B**) and P2 (**C**) and metallopeptides M1 (**D**) and M2 (**E**) at a concentration of 160 µmol L^−1^. Images were captured using an inverted microscope at 4× magnification.

All subsequent experiments were carried out only with the clinical isolate C19. First, we compared the four peptides to evaluate whether these compounds were able to decrease hyphal formation. As shown in Figure 6, untreated cells displayed the typical filamentous morphology, with evident germ tube formation, confirming the ability of the strain to undergo yeast-to-hypha transition under inducing conditions. Treatment with the tested compounds reduced germ tube formation to different extents. In particular, cells exposed to peptides showed a partial impairment of filamentation, with fewer elongated structures compared with the untreated control. A stronger inhibitory effect was observed after treatment with metallopeptides, where cells appeared mainly in yeast-like or poorly elongated forms, indicating a marked reduction in germ tube development.

### 3.5. Characterization of Antibiofilm Activity of Peptides

**Figure 7:**
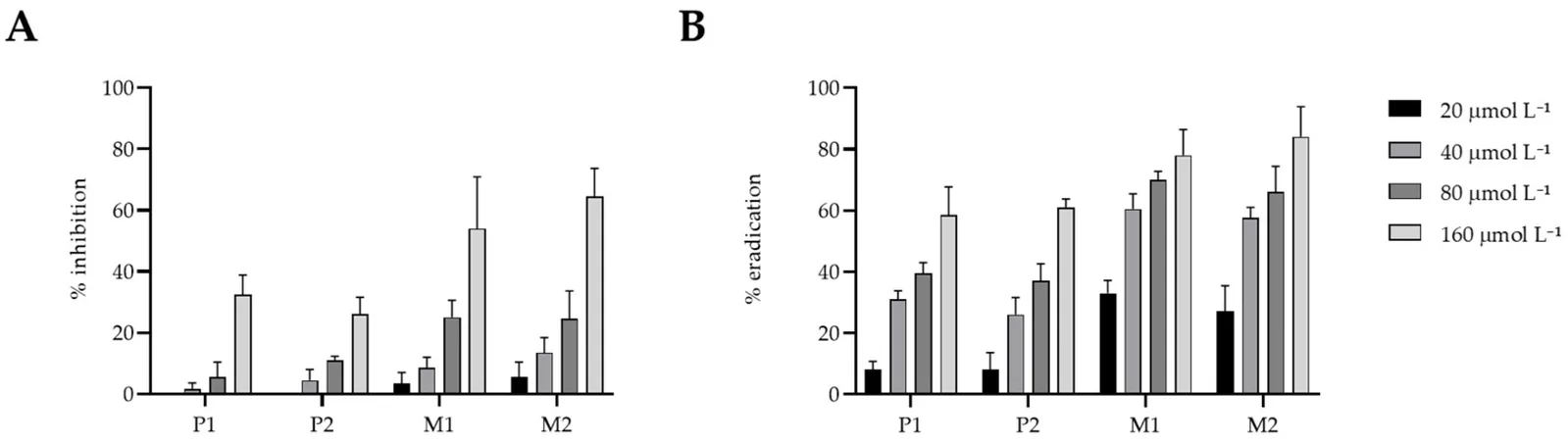
Antibiofilm activity of peptides and metallopeptides against *C. albicans* C19. (**A**) Inhibition of biofilm formation and (**B**) reduction in pre-established biofilm biomass after treatment with peptides P1 and P2 and metallopeptides M1 and M2 at concentrations ranging from 20 to 160 µmol L^−1^.

The clinical isolate C19 has been previously screened for biofilm formation spectrophotometrically at 570 nm and classified as a strong strain-forming biofilm (OD ˃ 0.652) [47]. The antibiofilm activity of the two peptides and the two metallopeptides against C19 was characterized by the CV method and percentages of biofilm inhibition are presented in Figure 7A,B for inhibition and eradication respectively. All compounds at sub-MICs (ranging from 20 to 160 µmol L^−1^) significantly inhibited the biofilm’s total biomass of C19 in a dose-dependent manner. As shown in Figure 7A, the highest reduction was observed at 160 µmol L^−1^, with inhibition percentages of approximately 30% for P1 and P2, and of about 50–60% for M1 and M2.

As reported in Figure 7B, peptides and metallopeptides demonstrated concentration-dependent biofilm eradication activity against existing C19 biofilms. The highest dose of both functionalized peptides of 160 µmol L^−1^ had a significant eradication effect, eliminating pre-formed biofilms by about 80%. Compared with its original drug, M1 and M2 demonstrated much higher levels of deleterious activity against established biofilms than P1 and P2 that reached only about 60% of eradication at the same concentration.

### 3.6. Persistent Biofilm Formation

**Figure 8:**
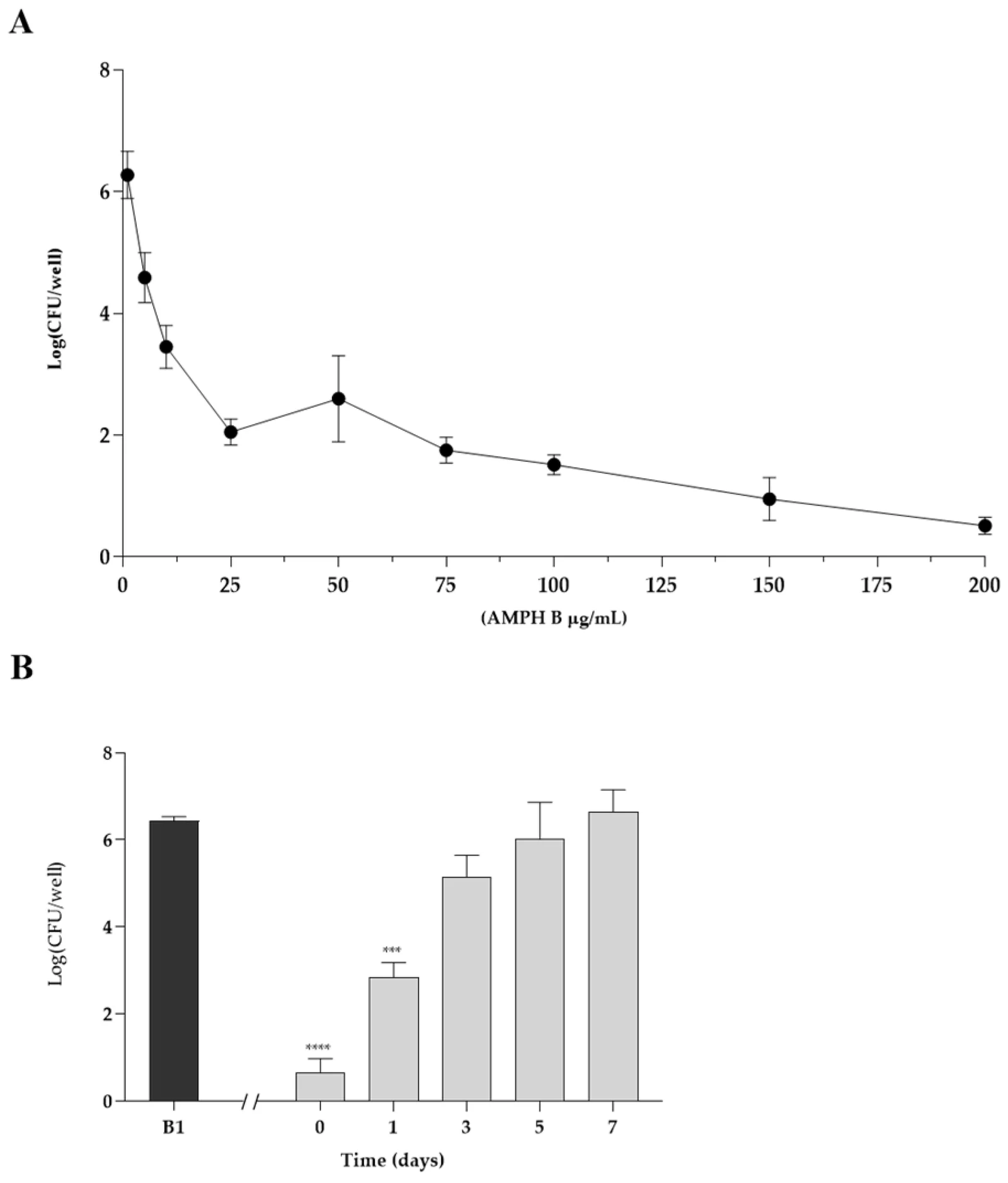
Detection of persistent of *C. albicans* C19 biofilm after treatment with Amphotericin B. Results are represented as mean of Log(CFU/mL); error bars represent standard deviation (**A**). Secondary biofilm development from *C. albicans* C19 persister-derived cells; *** *p* < 0.001, **** *p* < 0.0001 (Dunnett’s test) (**B**).

Amph B was used to challenge the primary biofilm formed by *C. albicans* C19 on a polystyrene surface. The established *C. albicans* C19 biofilm was exposed to increasing concentrations of Amph B, and, for each concentration, the number of viable residual biofilm-associated cells was determined as Log(CFU/mL). As shown in Figure 8A, Amph B treatment induced a marked reduction in biofilm-associated viable cells, with the fungal load decreasing from approximately 6 Log(CFU/mL) in the untreated control to about 2 Log(CFU/mL) after exposure to 25 µg/mL Amph B. However, further increases in Amph B concentration did not result in complete eradication of the biofilm population. Indeed, residual viable cells were still detected even at the highest concentrations tested, reaching approximately 0.5 Log(CFU/mL) at 200 µg/mL.

To further confirm the ability of the surviving cells to resume growth, Amph B-tolerant cells recovered from the primary biofilm were used to evaluate the development of a secondary biofilm over time. As shown in Figure 8B, persister-derived cells were able to re-establish a new biofilm on the polystyrene surface. At time 0, immediately after antifungal treatment, only a very low number of viable cells was detected, with values below 1 Log(CFU/well). However, a progressive increase in viable biofilm-associated cells was observed during incubation, reaching approximately 3 Log(CFU/well) after 1 day and more than 5 Log(CFU/well) after 3 days.

At later time points, the number of viable cells further increased, reaching about 6 Log(CFU/well) after 5 days and approximately 6.5 Log(CFU/well) after 7 days.

### 3.7. Persistent Biofilm Eradication

**Figure 9:**
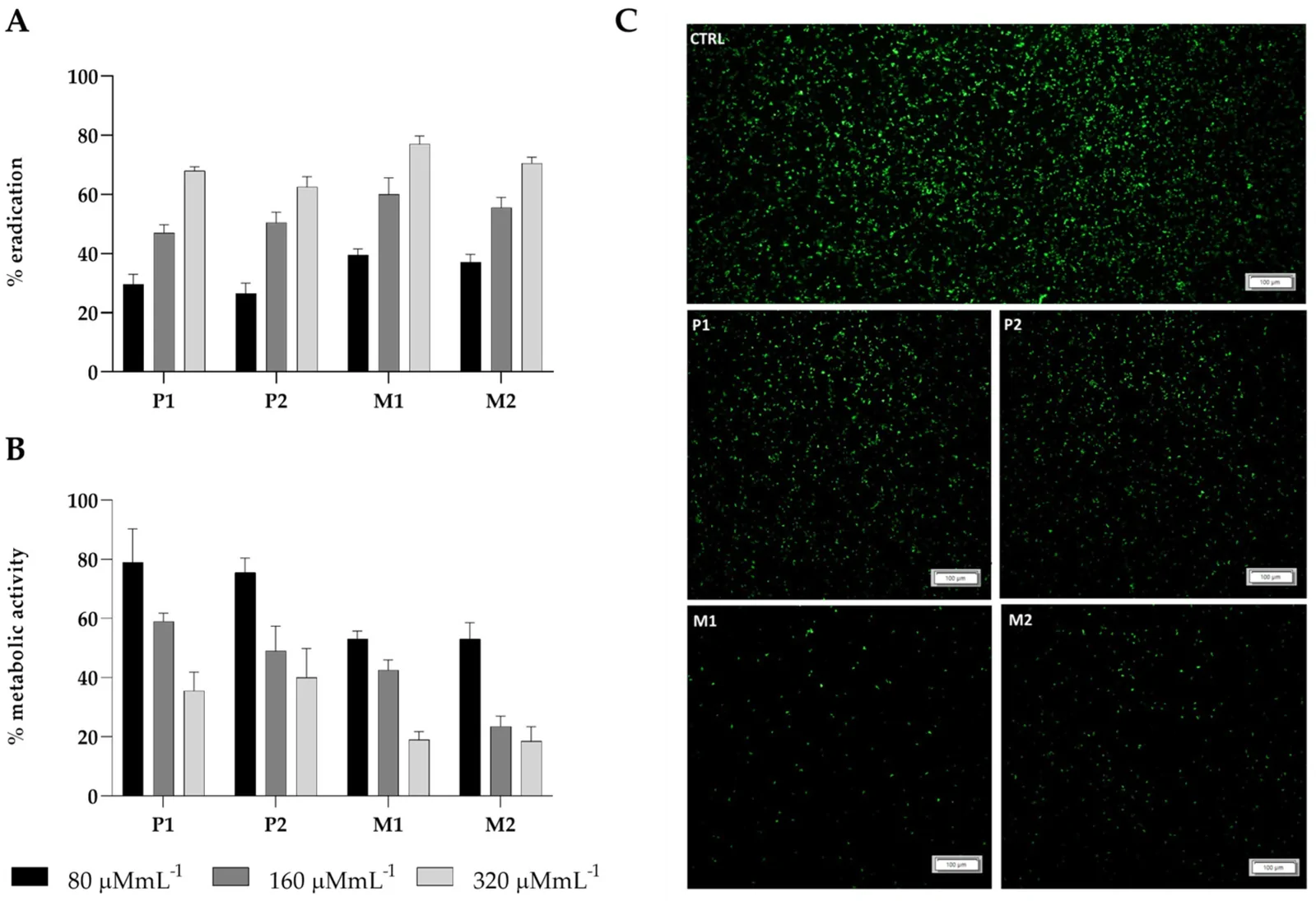
Effect of peptides and metallopeptides on *C. albicans* C19 persister-derived secondary biofilms tested at concentrations of 80, 160, and 320 µmol L^−1^. (**A**) Percentage of total biofilm biomass eradication, evaluated by CV staining. (**B**) Percentage of residual metabolic activity of treated biofilms. Data are expressed as mean ± standard deviation. (**C**) Fluorescence microscopy analysis of *C. albicans* C19 persister-derived secondary biofilms following treatment with 160 µmol L^−1^ of peptides and metallopeptides and subsequently stained with SYTO 9.

The activity of peptides P1 and P2 and metallopeptides M1 and M2 against *C. albicans* C19 persister-derived secondary biofilms was evaluated by assessing both total biofilm biomass and metabolic activity, together with biofilm-associated cell distribution by fluorescence microscopy (Figure 9).

Regarding total biomass, all treatments induced a concentration-dependent eradication of secondary biofilms (Figure 9A). At 80 µmol L^−1^, biofilm biomass reduction ranged from approximately 27% to 39%, with M1 and M2 showing slightly higher activity than P1 and P2. Increasing the concentration to 160 µmol L^−1^ enhanced the eradication effect, reaching approximately 47% for P1, 51% for P2, 60% for M1, and 56% for M2. The highest activity was observed at 320 µmol L^−1^, where biofilm biomass eradication reached about 68% for P1, 63% for P2, 77% for M1, and 70% for M2.

Consistently, the metabolic activity of persister-derived secondary biofilms was reduced after treatment (Figure 9B). At 80 µmol L^−1^, residual metabolic activity remained relatively high for P1 and P2, whereas M1 and M2 showed a stronger reduction. At 160 µmol L^−1^, metabolic activity decreased to approximately 59% for P1, 49% for P2, 42% for M1, and 23% for M2. At the highest concentration tested, the most pronounced reduction was observed for the metallopeptides, particularly M1 and M2, which reduced residual metabolic activity to about 19% and 18%, respectively.

Fluorescence microscopy analysis following SYTO 9 staining further supported the quantitative findings (Figure 9C). Untreated persister-derived secondary biofilms were characterized by a dense and widely distributed population of fluorescent cells. Treatment with P1 and P2 reduced the density of biofilm-associated cells, although numerous fluorescent cells remained detectable. In contrast, biofilms treated with M1 and M2 appeared markedly less dense, with fewer and more dispersed fluorescent cells.

### 3.8. Gene Expression Modulation in C. albicans C19 Secondary Biofilms

**Figure 10:**
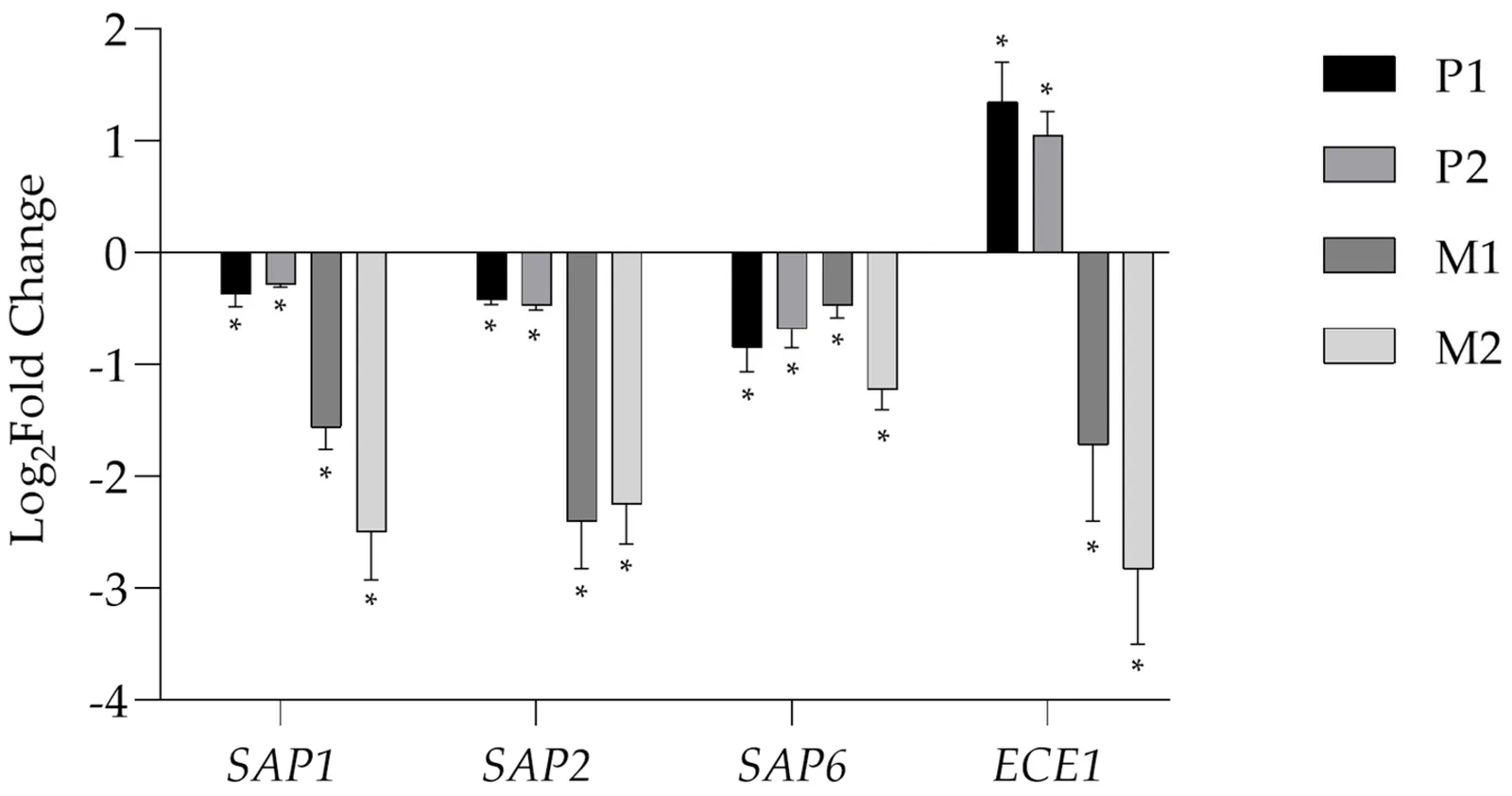
Expression analysis of selected genes of *C. albicans* C19 persistent biofilm using real-time qPCR (*SAP1*, *SAP2*, *SAP6* and *ECE1*) in response to peptides (P1, P2) and metallopeptides (M1, M2) at a concentration of 160 μmol L^−1^. Statistical significance versus the untreated control (log_2_FC = 0) was assessed using a two-tailed one-sample t-test with Holm–Šídák correction for multiple comparisons within each gene (* *p* < 0.05).

The expression of selected virulence-related genes was evaluated in secondary biofilms derived from *C. albicans* C19 persister cells after treatment with peptides P1 and P2 and metallopeptides M1 and M2. As shown in Figure 10, all treatments modulated the expression of *SAP1*, *SAP2*, *SAP6*, and *ECE1*, although with different intensity depending on the compound tested.

For *SAP1*, peptides P1 and P2 induced only a slight reduction in gene expression, whereas metallopeptides, particularly M2, produced a more evident downregulation. A similar trend was observed for *SAP2*, whose expression was only moderately reduced by P1 and P2, while M1 and M2 markedly decreased its expression. The *SAP6* gene was also downregulated by all compounds, with the strongest effect observed following treatment with M2.

A different expression profile was observed for *ECE1*. Treatment with peptides P1 and P2 slightly increased *ECE1* expression compared with the untreated secondary biofilm control. Conversely, metallopeptides M1 and M2 reduced *ECE1* expression, with M2 showing the most pronounced inhibitory effect.

### 3.9. Effect of Peptides and Metallopeptides on C. elegans Survival

The in vivo toxicity of peptides P1 and P2 and metallopeptides M1 and M2 was evaluated using *C. elegans* as a model organism. Nematodes were exposed to each compound, and survival was monitored over 72 h.

**Figure 11:**
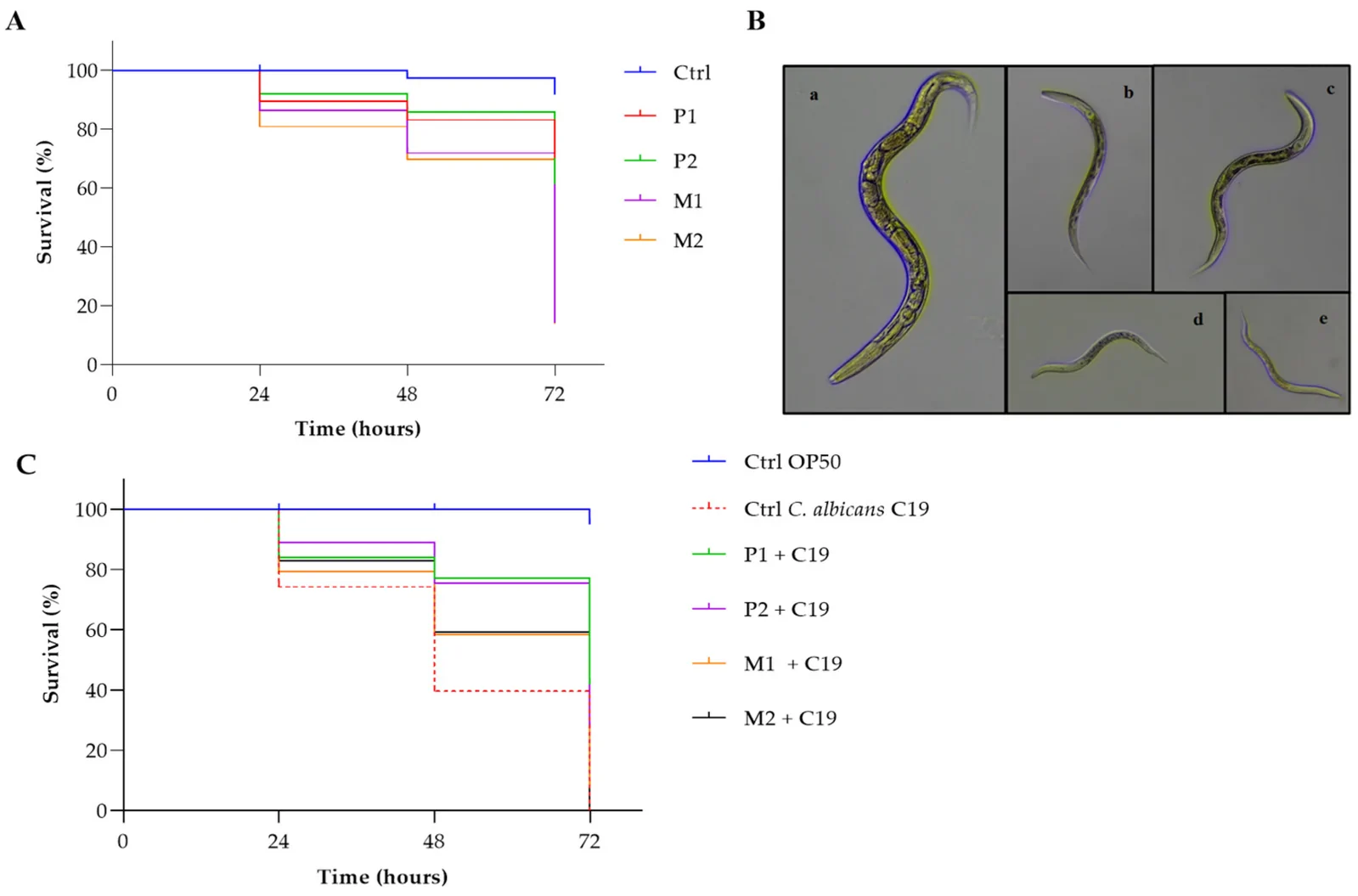
(**A**) Survival curves of *C. elegans* (wild-type N2 Bristol variant strain) exposed to peptides and metallopeptides. Nematodes were treated with peptides P1 and P2 or metallopeptides M1 and M2 (160 μmol L^−1^), and survival was monitored over 72 h. Untreated nematodes were used as control. Data are expressed as percentage of surviving worms over time. (**B**) Toxicity assay with *C.s elegans* after 72 h of exposure under different experimental. (**a**) Negative control (K-medium); (**b**) P1 and (**c**) P2; (**d**) M1 and (**e**) M2. (**C**) Survival curves of *C. elegans* larvae infected with *C. albicans* C19 and treated with peptides and metallopeptides 160 μmol L^−1^ for 24, 48 and 72 h.

As shown in Figure 11A, the untreated control group maintained high survival throughout the experiment, with only a slight decrease observed at 72 h. After 24 h of exposure, all treated groups showed a moderate reduction in survival, although values remained above 80%. In particular, survival was approximately 89% for P1, 91% for P2, 86% for M1, and 81% for M2.

At 48 h, survival remained relatively stable for P1 and P2, with values around 83–85%, whereas a more evident decrease was observed for the metallopeptides, particularly M1 and M2, whose survival values were approximately 72% and 70%, respectively.

At the final time point, P1 and P2 showed a better safety profile, with survival values remaining above 80% for P1 and approximately 60% for P2. Conversely, prolonged exposure to metallopeptides resulted in a stronger reduction in nematode survival, with M1 and M2 decreasing survival to approximately 14–15% at 72 h.

To evaluate the potential toxic effects of the tested compounds on *C. elegans* development and reproduction, nematodes were monitored after 72 h of exposure (Figure 11B). The untreated control (a) clearly highlights the normal presence of eggs within the adult body. Conversely, distinct physiological alterations were observed among the treated groups. Nematodes exposed to both peptide treatments (P1 and P2) successfully reached the adult stage; however, they completely failed to exhibit reproductive capacity, showing a total absence of eggs within their bodies compared to the control group (b,c). In contrast, organisms treated with the complexes (M1 and M2) failed to transition to adulthood, remaining arrested at the early larval stages (L2–L3) (d,e), and consequently showing no egg production. These phenotypic observations indicate that while the complexes exert toxicity by restricting development to early larval stages, the peptides might alter reproduction physiology in adulthood.

### 3.10. Infection Assay with C. elegans

The protective effect of peptides P1 and P2 and metallopeptides M1 and M2 was evaluated in *C. elegans* infected with *C. albicans* C19. Nematodes maintained on *E. coli* OP50, used as the standard food source, were included as the non-infected control. As shown in Figure 11A, this control group maintained almost complete survival throughout the experiment.

In contrast, infection with *C. albicans* C19 markedly reduced nematode survival over time. The infected untreated control showed a progressive decline in survival, decreasing from approximately 75% at 24 h to about 40% at 48 h, with almost complete mortality observed at 72 h.

Treatment with peptides and metallopeptides partially improved nematode survival compared with the infected untreated control. At 24 h, all treated groups showed higher survival than the infection control, with values ranging from approximately 79% to 89%. At 48 h, P1 and P2 displayed the best protective effect, maintaining survival at approximately 76–77%, whereas M1 and M2 showed lower survival values, around 59–62%.

At the final time point, P1 and P2 still provided partial protection, with survival values higher than those observed in the infected untreated control. In particular, P1 showed the most favorable profile, maintaining approximately 40% survival at 72 h, followed by P2. Conversely, metallopeptides M1 and M2 showed a weaker protective effect at prolonged exposure, with survival markedly decreasing at 72 h.

## 4. Discussion

Antimicrobial peptides are promising alternatives or complements to conventional antifungals because of their broad-spectrum activity, rapid mode of action, and reduced propensity to induce stable resistance compared with conventional drugs [9,10]. Among naturally occurring AMPs, Histatin 5 (Hst5) is one of the best-characterized salivary peptides displaying potent activity against *C. albicans*. Unlike membrane-lytic AMPs, Hst5 exerts its antifungal activity through a multifactorial intracellular mechanism. Following binding to fungal cell-wall components and internalization via specific transporters, Hst5 induces ATP leakage, ionic imbalance, mitochondrial dysfunction, oxidative stress, and ultimately fungal cell death [52,53,54,55]. Nevertheless, the therapeutic application of Hst5 remains limited by its susceptibility to proteolytic degradation by secreted aspartyl proteases (Saps) and by the reduced activity observed under physiological environmental conditions [23,35]. Recent engineering approaches have demonstrated that structural optimization of Histatin 5-derived peptides may improve antifungal selectivity while preserving the multifaceted intracellular mechanism of action [22].

To overcome these limitations, two complementary strategies were explored in the present study. First, lysine residues susceptible to proteolytic cleavage were replaced with arginine to generate peptide P2. Arginine conserves the positive charge required for interaction with negatively charged fungal surfaces while increasing resistance to Sap-mediated degradation [23,24]. Second, both peptides were functionalized with Ag(I), generating metallopeptides M1 and M2. Metal coordination has attracted increasing attention as a strategy to improve antimicrobial peptide performance because it combines the biological properties of the peptide scaffold with the intrinsic antimicrobial activity of transition metals, frequently resulting in enhanced stability, altered molecular conformation, and improved antimicrobial efficacy [21,27].

In the present study, the biological behavior of the native Hst5-derived fragment P1 and its arginine-substituted analog P2 was therefore compared with that of the corresponding Ag(I)-functionalized metallopeptides M1 and M2.

Physicochemical analyses confirmed successful peptide synthesis and Ag(I) functionalization. Silver binding induced marked conformational changes, converting the α-helical structure of the unconjugated peptides into a different conformation, which may contribute to the distinct biological activity of the metallopeptides, substantially improving antifungal activity.

Whereas P1 and P2 inhibited only the reference strain *C. albicans* ATCC 90028 and showed limited activity against the clinical isolate C19, both metallopeptides displayed identical MIC and MFC values against both strains. The divergence observed between MIC (320 µmol L^−1^) and MFC (640 µmol L^−1^) values for the metallopeptides indicates a predominantly fungistatic profile at lower doses, shifting to fungicidal activity only at higher concentrations. This behavior reflects their multi-target mode of action, where sub-MICs are nonetheless sufficient to disrupt vital morphogenetic pathways, such as the hyphal transition, which directly correlates with their strong antibiofilm and anti-virulence efficacy. This finding suggests that Ag(I) functionalization partially overcomes strain-dependent mechanisms, reducing susceptibility to Hst5-derived peptides. Silver ions are known to interact simultaneously with multiple cellular targets, including membrane proteins, thiol-containing enzymes, respiratory pathways, DNA replication and oxidative homeostasis, thereby generating antimicrobial effects through several complementary mechanisms [36,37]. Such multi-target activity may explain why the clinical isolate remained susceptible to the metallopeptides despite its reduced sensitivity to the unconjugated peptides.

An interesting observation emerging from this study is that significant antibiofilm effects were detected at concentrations well below the MIC values. This behavior is increasingly recognized among AMPs, whose antibiofilm activity frequently results from interference with adhesion, quorum sensing, extracellular matrix production and morphogenesis rather than direct fungicidal activity [56]. Both peptides reduced biofilm formation and partially disrupted mature biofilms, whereas Ag(I) functionalization consistently enhanced both preventive and eradication activity. Since crystal violet staining quantifies total adherent biomass without distinguishing viable from dead cells, these results should be interpreted as structural disruption of the biofilm rather than complete elimination of biofilm-associated fungal cells [57,58]. This finding is particularly relevant because established *Candida* biofilms are less susceptible to conventional antifungals due to extracellular matrix protection, altered metabolism, stress responses, and phenotypic heterogeneity [59,60].

The inhibition of hyphal development provides a plausible mechanistic explanation for the observed antibiofilm effects. Hyphal formation represents a fundamental virulence determinant of *C. albicans*, facilitating tissue invasion, biofilm maturation and immune evasion [59,61]. Microscopic observations demonstrated that both peptides reduced germ tube formation, while metallopeptides almost completely suppressed filamentation. Since mature biofilm architecture largely depends on hyphal development, the stronger inhibition of filamentation induced by M1 and M2 likely contributed to their superior antibiofilm activity. Furthermore, inhibition of morphogenesis may attenuate the expression of multiple downstream virulence determinants, thereby reducing fungal pathogenicity beyond simple growth inhibition. One of the most clinically relevant aspects of the present study is the evaluation of amphotericin B persister-derived biofilms. Persister cells represent a transient phenotypic variant capable of surviving lethal antimicrobial concentrations without acquiring stable genetic resistance [62]. Their persistence is considered one of the major causes of chronic and recurrent fungal infections, including recurrent vulvovaginal candidiasis [63,64]. The biphasic killing curve observed after amphotericin B treatment, followed by survival of a small residual population despite increasing drug concentrations, closely resembles the classical persister phenotype previously described in *Candida* biofilms [40]. Importantly, these surviving cells retained the capacity to regenerate mature secondary biofilms, confirming that the residual population remained viable and highly tolerant. The surviving cells retained the ability to repopulate the surface and generate a mature secondary biofilm, reaching approximately 6.5 Log CFU/well after seven days. This behavior supports the role of phenotypically tolerant cells in infection persistence and recurrence after antifungal treatment [40,62], a mechanism that may be particularly relevant in recurrent vulvovaginal candidiasis, where biofilm growth contributes to reduced drug susceptibility and prolonged fungal persistence [63,64].

Importantly, both peptides and metallopeptides reduced the biomass of secondary biofilms generated from amphotericin B-surviving cells in a concentration-dependent manner, indicating that the persister-derived phenotype did not confer complete protection against Hst5-based compounds. This is consistent with previous evidence supporting antimicrobial peptides as promising tools against *C. albicans* persister-derived biofilms [40]. Treatment, particularly with M1 and M2, downregulated SAP1, SAP2, SAP6, and ECE1, genes involved in epithelial invasion, hyphal development, biofilm maturation, and host tissue damage. Notably, the decreased ECE1 expression is consistent with reduced candidalysin production and impaired hyphal differentiation. Together, these findings suggest that metallopeptides target not only fungal growth but also key virulence mechanisms [23,65,66,67].

However, the improved antimicrobial activity of the metallopeptides was accompanied by increased toxicity toward both mammalian cells and *C. Elegans.* At 320 µmol L^−1^, M1 and M2 showed the strongest antibiofilm activity but also marked HaCaT cytotoxicity, highlighting a clear activity–toxicity trade-off and limiting their immediate therapeutic applicability. While the unconjugated peptides displayed lower antifungal activity in vitro, they showed better cytocompatibility and provided more sustained protection in the in vivo infection model. These observations highlight the importance of evaluating antimicrobial candidates in integrated experimental systems that combine efficacy, virulence modulation, and host toxicity rather than relying exclusively on in vitro antifungal activity [68].

The mechanisms underlying the toxicity of M1 and M2 remain to be clarified. Since the present study did not distinguish between the biological effects of the intact metallopeptides and those potentially resulting from Ag(I) release, further investigations comparing metallopeptides, unconjugated peptides, and Ag(I) alone will be required. Likewise, direct quantification of fungal burden in infected nematodes and optimization of metallopeptide dosing or delivery strategies will be essential to determine whether antifungal efficacy can be maintained while minimizing host toxicity [69].

The *C. elegans* assays showed that the greater antifungal activity obtained through Ag(I) functionalization was accompanied by increased toxicity in vivo. Although the metallopeptides were more active against *C. albicans* in vitro, P1 and P2 showed lower toxicity and provided more sustained protection in infected nematodes. This comparison between antifungal activity and toxicity is particularly relevant in *C. elegans*-based antifungal screening, since this model allows both parameters to be evaluated under the same experimental conditions [70,71].

The effects on development and reproduction provided information that was not obtained from the survival assay alone. The absence of eggs in nematodes treated with P1 and P2 suggests a possible effect on reproduction, whereas the larval arrest observed following treatment with M1 and M2 indicates a stronger effect on development. Development and reproduction are commonly evaluated as sublethal toxicity endpoints in *C. elegans* and can provide information that is not obtained from survival analysis alone [72,73]. Specific reproductive assays are therefore required to confirm the effects of P1 and P2. In contrast, the larval arrest caused by M1 and M2 indicates that the toxicity of these compounds is not limited to their effect on nematode survival.

The greater toxicity of M1 and M2 may be related, at least in part, to the presence of Ag(I). Previous studies have shown that exposure to silver-containing materials can reduce growth, fertility and reproduction and increase ROS levels in *C. elegans* [69]. Silver nanoparticle exposure has also been associated with germ-cell damage and impaired reproductive integrity [74]. However, these studies examined silver nanoparticles rather than Ag(I)-coordinated peptides and were conducted under different experimental conditions. It is therefore not possible to determine from the present results whether the toxicity of M1 and M2 was caused by the complete metallopeptides or by the release of Ag(I). Further experiments comparing the metallopeptides with the corresponding unconjugated peptides and an Ag(I) control are required to clarify this point.

In the infection assay, P1 and P2 provided more sustained protection than M1 and M2. This finding suggests that the lower antifungal activity of the unconjugated peptides in vitro was accompanied by an advantage in vivo because of their lower toxicity. In contrast, the reduction in survival observed following prolonged treatment with M1 and M2 may reflect both the progression of the infection and the toxicity of the metallopeptides. Since hyphal formation is an important determinant of *C. albicans* virulence in the *C. elegans* infection model, the protective effect observed in the present study may be related, at least in part, to the inhibition of germ-tube formation observed in vitro [70,75]. The modulation of virulence-related genes is consistent with this interpretation, but these genes were not evaluated in infected nematodes and their contribution to the protective effect cannot be confirmed from the present results.

Fungal viability was also not measured directly in infected nematodes. Therefore, it remains to be established whether treatment reduced the fungal population in vivo. Further experiments should evaluate fungal viability in infected nematodes and determine whether lower concentrations of M1 and M2 can maintain antifungal activity while reducing toxicity.

## 5. Conclusions

Overall, the present study demonstrates that Ag(I) functionalization enhances the antifungal, antibiofilm, and anti-virulence activity of Hst5-derived peptides, extending their efficacy to secondary biofilms generated by *C. albicans* persister cells, a clinically relevant model of recurrent vulvovaginal candidiasis. However, the increased biological activity was accompanied by higher host toxicity, indicating that further optimization of peptide selectivity and delivery strategies is required. Despite this limitation, these findings support Hst5-derived metallopeptides as promising candidates for the development of novel therapeutic approaches targeting persister-associated biofilms and recurrent *C. albicans* infections.

## Figures and Tables

**Table 1:** Primary structure of the Hst5, 14 Hst5 and 14 Hst5 K11,17R peptides, demonstrating the origin of the fragment in the original peptide and the amino acid residue modifications in the fragment.

Peptide	Primary Structure
*Hst5*	D S H A K R H H G Y K R K F H E K H H S H R G Y
*14 Hst5*	W K R K F H E K H H S H R G Y
*14 Hst5 K11,17R*	W R R K F H E R H H S H R G Y

**Table 2:** Theoretical and experimental mass/charge ratios by mass spectrometry analysis for the synthesized peptides.

Peptide	Charge (z)	Theoretical *m*/*z*	Experimental *m*/*z*	Theoretical Molar Mass (g mol^−1^)	Experimental Molar Mass (g mol^−1^)
*14 Hst5*	3	678.75	678.72	2033.26	2032.72
	4	509.31	509.07		
*14 Hst5 K11,17R*	3	697.43	697.40	2089.29	2088.72
	4	523.32	523.06		

**Table 3:** Theoretical and obtained molar mass from MALDI-TOF mass spectrometry analysis for the metallopeptides.

Metallopeptide	Charge (z)	Theoretical Molar Mass (g mol^−1^)	Experimental Molar Mass (g mol^−1^)
*14 Hst5–Ag*	1	2141.13	2139.92
*14 Hst5 K11,17R–Ag*	1	2197.16	2195.98

**Table 4:** Minimal inhibitory concentration (MIC) and minimum fungicidal concentration (MFC) of peptides (P1, P2) and metallopeptides (M1, M2) against *C. albicans* ATCC 90028 and C19.

	P1		P2		M1		M2	
	MIC	MFC	MIC	MFC	MIC	MFC	MIC	MFC
*C. albicans* ATCC 90028	640	>640	640	>640	320	640	320	640
*C. albicans* C19	>640	>640	>640	>640	320	640	320	640

## Data Availability

The original contributions presented in this study are included in the article/Supplementary Materials. Further inquiries can be directed at the corresponding author.

## Supplementary Materials

The following supporting information can be downloaded at https://www.mdpi.com/article/10.3390/microorganisms14102304/s1, Figure S1: Experimental flowchart of the study; Table S1: Primer sequences used in this study.
