# Supplementary material for: Histatin-Based Metallopeptides Target Secondary Biofilms Derived from Candida albicans Persister Cells in Recurrent Vulvovaginal Candidiasis
Source: Microorganisms. 2026 Oct 10;14(10):2304. doi: 10.3390/microorganisms14102304 (PMC13707523; doi:10.3390/microorganisms14102304)
Supplement: Supplementary file 1 [file microorganisms-14-02304-s001.zip › microorganisms-4597719-supplementary.pdf]

**Figure S1.** Experimental flowchart of the study.

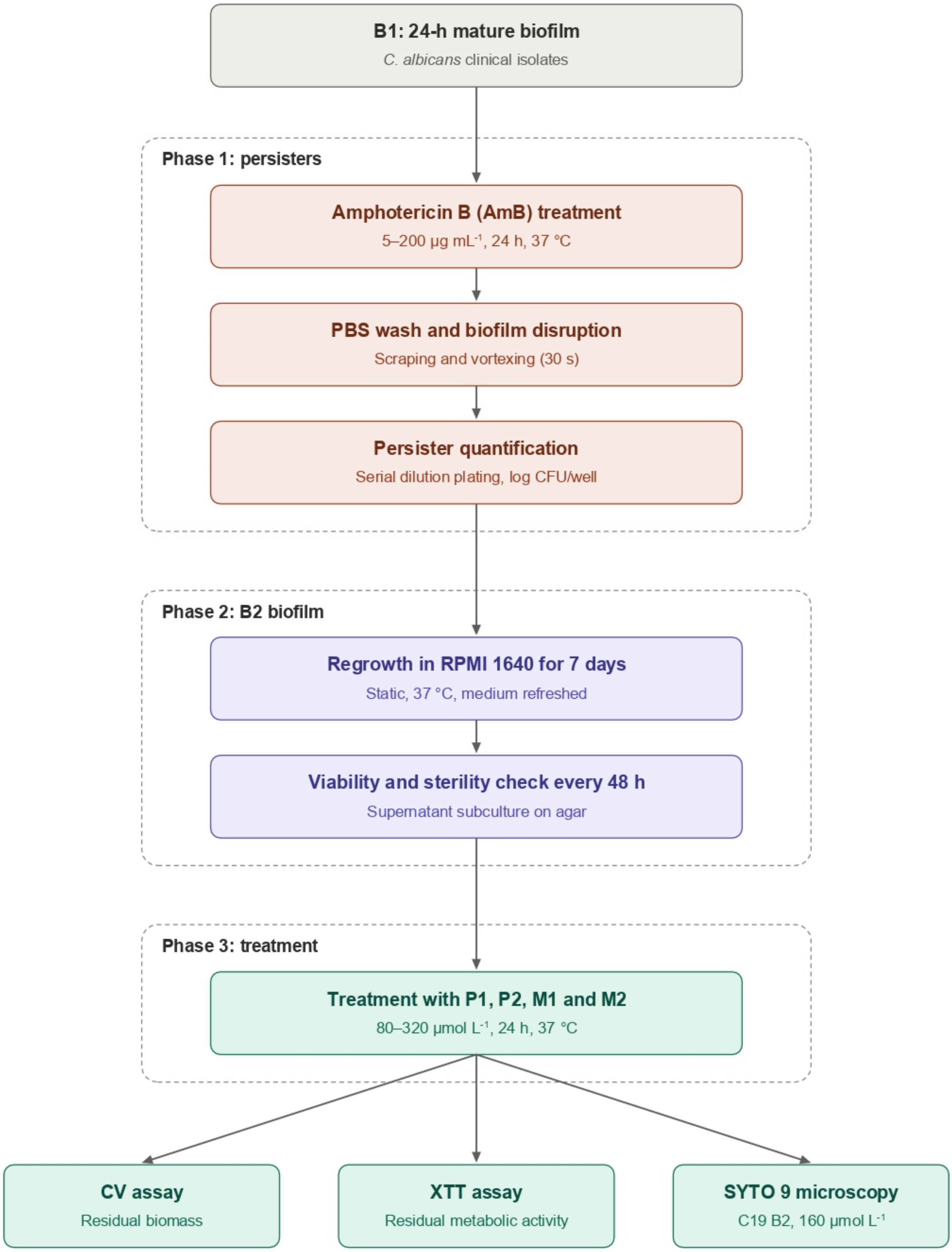

**Table S1.** Primer sequences used in this study

| Gene name | Acronym | Primer Name | Sequence (5′→ 3′) |
|---|---|---|---|
| *Secreted aspartyl proteinase 1* | *SAP_1* | SAP1_C.al_F<br>SAP1_C.al_R | TTCATCGCTCTTGCTATTGCTT<br>TGACATCAAAGTCTAAAGTGACAAAACC |
| *Secreted aspartyl proteinase 2* | *SAP_2* | SAP2_F<br>SAP2_R | TCCTGATGTTAATGTTGATTGTCAAG<br>TGGATCATATGTCCCCTTTTGTT |
| *Secreted aspartyl proteinase 6* | *SAP_6* | SAP6_C.al_F<br>SAP6_C.al_R | CCTTTATGAGCACTAGTAGACCAAACG<br>TTACGCAAAAGGTAACTTGTATCAAGA |
| *Actin* | *ACT1* | ACT1_F<br>ACT1_R | AGCCCAATCCAAAAGAGGTATT<br>GCTTGGGTCAACAAAACTGG |
| *Extent of Cell Elongation 1* | *ECE1* | ECE1_F<br>ECE1_R | ATCGAAAATGCCAAGAGAG<br>AGCATTTTCAATACCGACAG |
